# Supplementary material for: Role of the Scavenger Receptor CD36 in Accelerated Diabetic Atherosclerosis
Source: Int J Mol Sci. 2020 Oct 5;21(19):7360. doi: 10.3390/ijms21197360 (PMC7583063; doi:10.3390/ijms21197360)
Supplement: Supplementary file 1 [file ijms-21-07360-s001.pdf]

Supplementary material

Role of the scavenger receptor CD36 in accelerated diabetic atherosclerosis

Miquel Navas-Madroñal[1†], Esmeralda Castelblanco[3,4†], Mercedes Camacho[1,2], Marta Consegal[1], Anna Ramirez-Morros[5], Maria Rosa Sarrias[6], Paulina Perez[7], Nuria Alonso[4,5], María Galán[1,2*], Dídac Mauricio[3,4*].

Corresponding authors:

Dídac Mauricio
Department of Endocrinology & Nutrition
Hospital de la Santa Creu i Sant Pau
Sant Quintí, 89
08041 Barcelona, Spain
TEL: +34 93 556 56 61; FAX: 93 556 56 02
E-mail: didacmauricio@gmail.com

María Galán
Sant Pau Biomedical Research Institute (IIB Sant Pau)
Hospital de la Santa Creu i Sant Pau
Avda. Sant Antoni Maria Claret, 167
08025 Barcelona. Spain.
TEL: +34 93 556 56 22 / 5711; FAX: +34 93 556 55 59
E-mail: mgalana@santpau.cat

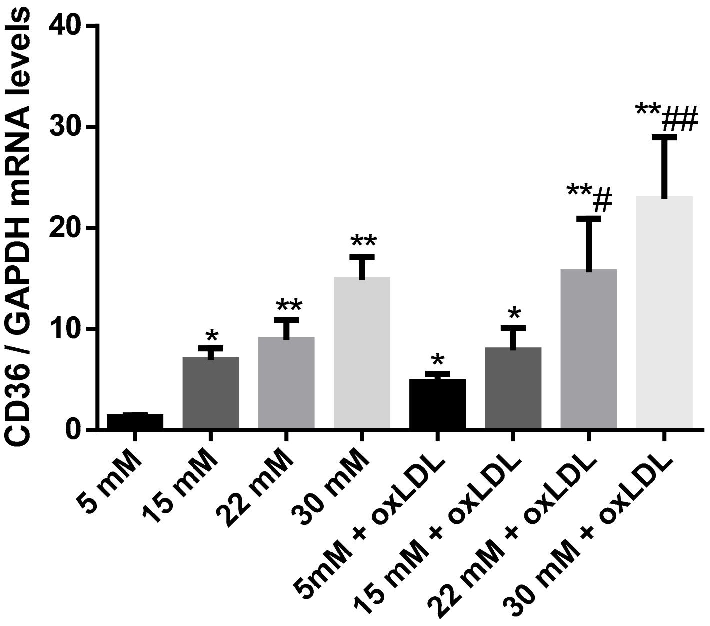

Figure Supplementary 1. CD36 expression in VSMC in response to increasing concentrations of glucose ± oxLDL. Starved VSMC were cultured for 72 h with M199 medium containing 5 mM (normal glucose, NG), 15 mM, 22 mM or 30 mM of glucose. CD36 mRNA levels were determined by quantitative real Time PCR analysis (qPCR) and normalized to GAPDH. Mean fold change relative to 5 mM (NG) ± SEM from four independent experiments performed in duplicate are shown. One-way ANOVA test was performed for statistical significance: **p*≤ 0.05 vs 5 mM; ***p*≤ 0.01 vs 5 mM; #*p*≤ 0.05 vs 22 mM; ##*p*≤ 0.01 vs 22 mM.

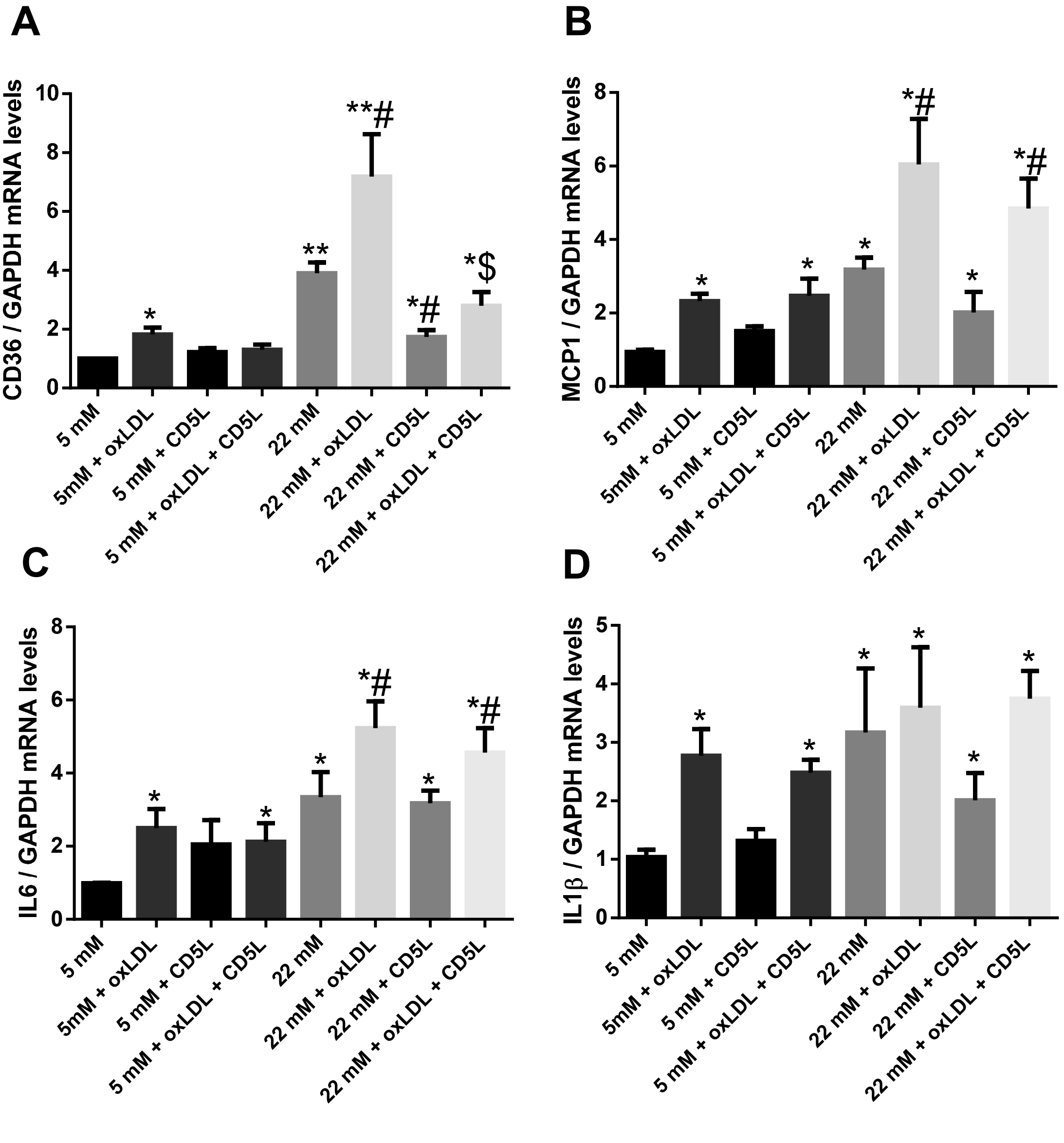

Figure Supplementary 2. Co-incubation of VSMC with recombinant human CD5L blunts CD36 induction but does not affect to inflammatory markers expression. **A-D)** CD36, MCP1, IL6 and IL-1β mRNA levels were determined by qPCR analysis and normalized to GAPDH after co-incubation with rCD5L for 48 h (N=5). *$p \leq 0.05$ vs 5 mM; **$p \leq 0.01$ vs 5 mM; #$p \leq 0.05$ vs 22 mM; \$$p \leq 0.05$ vs 22 mM+oxLDL.

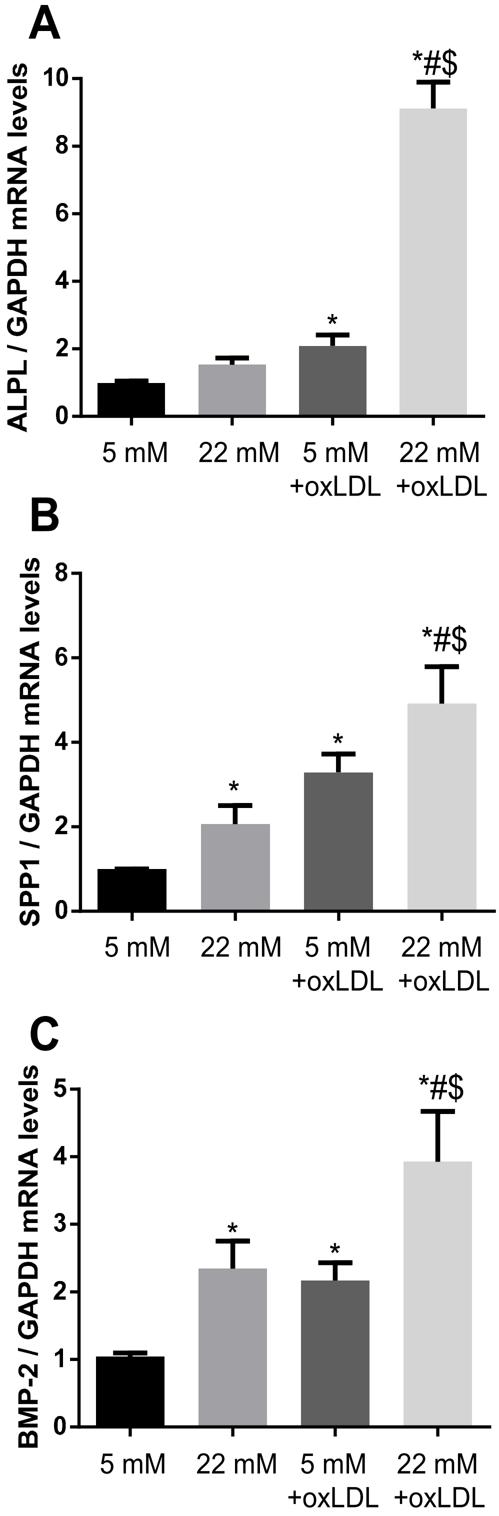

Figure Supplementary 3. Expression of calcification markers in VSMC exposed to HG ± oxLDL. The expression of bone matrix proteins that regulate the calcification process were induced in VSMC cultured with high glucose for 72 h. **A-C)** Alkaline phosphatase (ALPL), Secreted phosphoprotein 1 (SPP1) and Bone morphogenetic protein 2 (BMP2) mRNA levels were determined by qPCR analysis and normalized to GAPDH. Mean fold change relative to cultured VSMC in NG medium ± SEM (N=4). *$p \leq 0.05$ vs 5 mM; #$p \leq 0.05$ vs 22 mM; \$$p \leq 0.05$ vs 22mM+oxLDL.

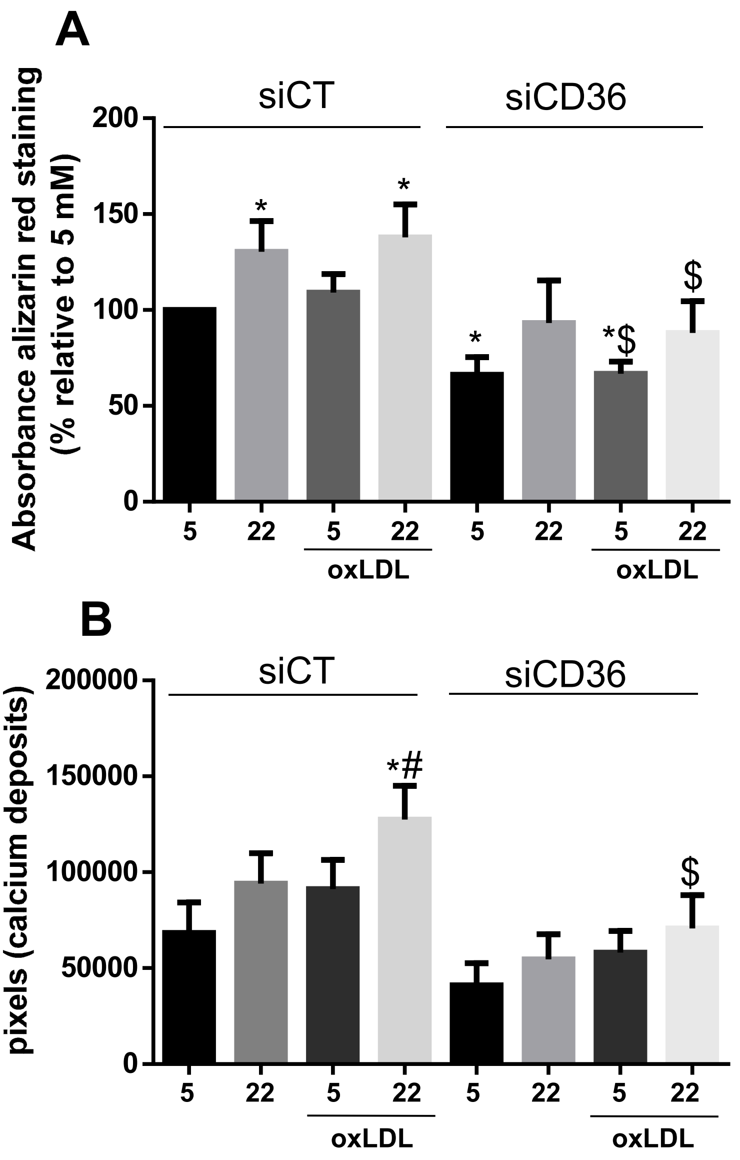

Figure Supplementary 4. VSMC were transfected with siRNA-CT or siRNA-CD36 and cultured in osteogenic medium with NG or HG ± oxLDL for 10 days. **A)** Quantification of alizarin red staining by colorimetry. **B)** Quantification of alizarin red staining by imaging analysis. Results are expressed as mean ± SEM (N=5). *p<0.05 *vs*. 5 mM siRNA-CT; #p<0.05 *vs* 22 mM siRNA-CT; $p<0.05 *vs*. 22 mM+oxLDL siRNA-CT.
